# The siRNA Off-Target Effect Is Determined by Base-Pairing Stabilities of Two Different Regions with Opposite Effects

**DOI:** 10.3390/genes13020319

**Published:** 2022-02-09

**Authors:** Yoshiaki Kobayashi, Shen Tian, Kumiko Ui-Tei

**Affiliations:** 1 Department of Biological Sciences, Graduate School of Science, The University of Tokyo, Tokyo 113-0033, Japan; yoshiaki-kobayashi@g.ecc.u-tokyo.ac.jp (Y.K.); shen.tian@u.nus.edu (S.T.); 2 Department of Computational Biology and Medical Sciences, Graduate School of Frontier Sciences, The University of Tokyo, Chiba 277-8561, Japan

**Keywords:** RNAi, siRNA, seed region, off-target effect, responsive region

## Abstract

In RNA interference (RNAi), small interfering RNA (siRNA) suppresses the expression of its target mRNA with a perfect complementary sequence. In addition, siRNA also suppresses the expression of unintended mRNAs with partially complementary sequences mainly within the siRNA seed region (nucleotides 2–8). This mechanism is highly similar to microRNA (miRNA)-mediated RNA silencing, and known as the siRNA-mediated off-target effect. Previously, we revealed that the off-target effect is induced through stable base-pairing between the siRNA seed region and off-target mRNAs, but not induced through unstable base-pairing. However, in our recent study, we found that the siRNA seed region consists of two functionally different domains: nucleotides 2–5, essential for off-target effects, and nucleotides 6–8, involved in both RNAi and off-target effects. In this study, we investigated the most responsible region for the off-target effect by conducting a comprehensive analysis of the thermodynamic properties of all possible siRNA subregions that involved a machine learning technique using a random sampling procedure. As a result, the thermodynamic stability of nucleotides 2–5 showed the highest positive correlation with the off-target effect, and nucleotides 8–14 showed the most negative correlation. Thus, it is revealed that the siRNA off-target effect is determined by the base-pairing stabilities of two different subregions with opposite effects.

## 1. Introduction

**Figure 1:**
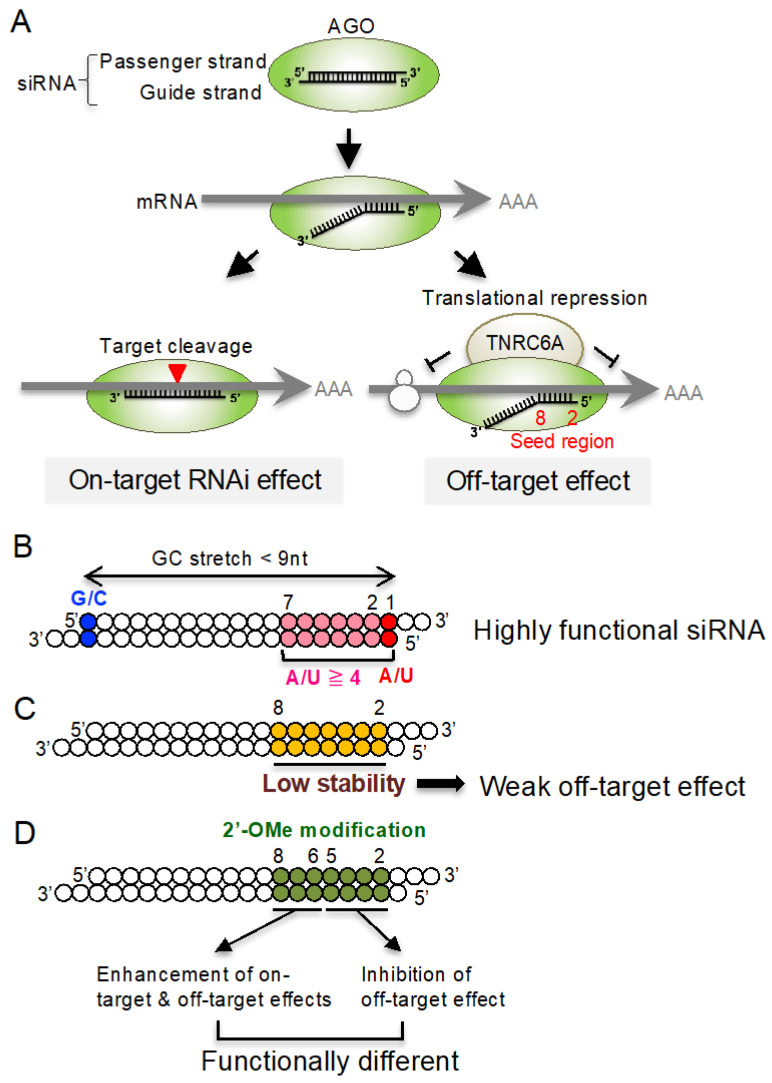
Mechanism of siRNA-mediated on-target RNAi and off-target effects. (**A**) The siRNA is composed of a double-stranded RNA of 21-nucleotide-long guide and passenger strands with 2-nucleotide 3′-overhangs. siRNA transfected into cells is loaded onto AGO2 protein. The siRNA guide strand initially base-pairs with its on-target and off-target mRNAs via sequence complementarity with the seed region. Subsequently, the guide strand RNA base-pairs with its on-target mRNA, which has an entirely complementary sequence, and AGO2 cleaves it to repress its expression. As a mechanism different from that for RNAi, the expression of off-target mRNAs with sequence complementarities with the siRNA seed region alone is reduced by off-target effects via a mechanism similar to miRNA-mediated translational repression. The TNRC6A protein associated with the AGO protein represses the translation of off-target mRNAs [16]. (**B**) Sequence rules of siRNAs predicted to be functional in mammalian cells: A/U at the 5′ end of the guide strand, G/C at the 5′ end of the passenger strand, more than 4 A/Us in the 5′ terminus 7-nucleotide of the guide strand, and no GC stretch longer than 8 nucleotides [17]. (**C**) siRNA sequence with weak off-target activity [18]. The siRNA with low *T_m_* value in the seed region (nucleotides 2–8) exhibits weak off-target effect. (**D**) The nucleotides in the seed region are functionally divided into two domains in response to 2′-OMe modifications: 2′-OMe modifications of nucleotides 2–5 inhibit the off-target effect of siRNA, whereas 2′-OMe modifications of nucleotides 6–8 promote both on-target and off-target effects [19].

RNA interference (RNAi) is a natural cellular process that affects post-transcriptional gene silencing in eukaryotic systems [1,2]. Small interfering RNA (siRNA) is a double-stranded RNA with 2-nucleotide 3′ overhangs [3,4,5,6]. The siRNA introduced into cell is loaded onto an Argonaute (AGO) protein, a core protein of the RNA-induced silencing complex (RISC) (Figure 1A) [7,8], and is unwound into two RNA strands: the guide strand and the passenger strand [9,10,11,12]. The guide strand base-pairs with its target mRNA, with a perfect complementary sequence, on the AGO protein, and the target mRNA is cleaved by AGO2 [13,14,15]. Thus, RNAi is widely recognized as not only a powerful research tool for functional genomics, but also as a promising candidate for therapeutic modalities.

Although most siRNAs are functional in *Caenorhabditis elegans* and *Drosophila*, a limited fraction of siRNAs are functional in mammalian cells [17]. We have revealed promising sequence rules of functional siRNAs in mammalian cells: (i) A/U at the 5’ end of the siRNA guide strand, (ii) G/C at the 5´ end of the passenger strand, (iii) four or more A/U residues in the 7-nucleotide 5´ terminus of the guide strand, and (iv) no G/C stretch of ≥9 nucleotides long (Figure 1B) [17]. The importance of these requirements has been verified in a number of studies, and more than 95% of siRNAs that simultaneously satisfy these four sequence conditions have been revealed to be functional. The asymmetry in the thermodynamic stabilities of both siRNA termini is indispensable for determining the direction of unwinding into single-stranded RNAs [20,21,22], and the easily unwound 5′-terminus is anchored within a binding pocket in the MID domain of the AGO protein. The binding affinity of terminal A or U in the pocket is 30-fold higher than that of either G or C [23]. Thus, an RNA strand with an unstable 5′ terminus is capable of acting as a functional guide RNA.

Using a mechanism different from that described above for RNAi, siRNA suppresses non-target transcripts with partial sequence complementarities. This phenomenon is called the off-target effect, and is considered to be an undesirable side effect in RNAi research and therapeutics. This mechanism is highly similar to microRNA (miRNA)-mediated RNA silencing. In the canonical miRNA biogenesis pathway, the stem–loop structured primary-miRNA (pri-miRNA) with flanking regions transcribed in the nucleus, is cleaved into precursor-miRNA (pre-miRNA) [24,25,26,27,28]. The pre-miRNA is transported from the nucleus to the cytoplasm, and its loop region is cleaved off by the enzyme Dicer to generate a miRNA duplex in collaboration with the trans-activation response (TAR) RNA binding protein (TRBP) [29,30]. The miRNA duplex loaded onto the AGO protein is then unwound into single-stranded miRNAs; one RNA strand remains on the AGO protein and acts as miRNA, the other RNA strand is discarded [12,31,32,33]. The mature miRNA on the AGO protein guides the RISC to target mRNAs that have sequence complementarities, mainly with the seed region (nucleotides 2–8) of the miRNA, in their 3′ untranslated regions (UTRs) [34,35,36,37]. Subsequently, AGO recruits the trinucleotide repeat containing six (TNRC6) proteins, a scaffold protein that tethers effector proteins to destabilize and translationally repress target mRNAs by decapping or deadenylation [16]. Thus, miRNA-mediated RNA silencing does not cleave the target transcripts, unlike siRNA-induced RNAi. In a similar manner, the off-target transcripts of siRNA have complementary sequences with the siRNA seed region [38,39,40,41,42,43]. The reason the siRNA and miRNA seed regions are involved in RNA silencing or the off-target effect is that the phosphates of the backbone of the seed nucleotides are stably immobilized on the quasi-helical structured surface of the AGO protein, serving as the entry or nucleation site [44,45]. However, we have shown that the off-target effect of siRNA does not always occur, even when the seed sequence is complementary to the transcript. The thermodynamic stability between the siRNA seed region and the off-target transcript has a high impact on off-target activity: the higher the seed-target base-pairing stability, the higher the off-target effect [18]. Thus, the siRNA that has a seed sequence with low thermodynamic stability was considered to be a suitable candidate for preventing the off-target effect (Figure 1C). However, we recently found that the siRNA seed region is able to divide into two functionally different regions: nucleotides 2–5 and 6–8 [19]. The 2′-*O*-Methyl (2′-OMe) modifications of nucleotides 2–5 act to reduce off-target activity due to steric hindrance without affecting on-target (RNAi) activity, whereas those at nucleotides 6–8 enhance both on-target and most off-target activities, probably due to its high binding stabilities with on-target/off-target transcripts (Figure 1D). The difference between nucleotides 2–5 and 6–8 is consistent with the structural analyses, in which the seed region of the single-stranded guide RNA on the human AGO protein organizes into a helical conformation. The base-stacking in the helical structure is interrupted by a kink between nucleotides 6 and 7 [45,46,47,48,49]. This kink is induced by the helix-7 domain of human AGO2; the helix-7 insertion between nucleotides 6 and 7 creates a steric barrier for the base-pairing of nucleotides 6–8 with target transcripts. Furthermore, the helix-7 shifts to dock into the minor groove of the guide–target duplex in stable pairing. Thus, nucleotides 2–5 are considered to remain stable and immobile on the AGO protein in both the single-stranded and double-stranded forms, but the conformation of nucleotides 6–8 is unstable and flexible and easily altered by helix-7. Therefore, the steric hindrance induced by 2′-OMe modifications of nucleotides 2–5, but not those of nucleotides 6–8, reduces the off-target effect. However, the detailed contributions of nucleotides at each subregion in the entire siRNA towards off-target effects are unknown. In this study, the impacts of the thermodynamic stabilities of all the possible nucleotide subregions within siRNA on the on-target and off-target effects were analyzed via a machine learning technique using a random sampling procedure.

## 2. Materials and Methods

### 2.1. siRNA

RNA oligonucleotides (the guide and passenger strands) of each siRNA were chemically synthesized (GenePharma) and annealed to form endogenous siRNA duplexes. The siRNA sequences are shown in Supplementary Table S1. 

### 2.2. Cell Culture

Human HeLa cells were cultured in Dulbecco’s Modified Eagle’s Medium (FUJIFILM Wako, Osaka, Japan) containing 10% fetal bovine serum (BioWest, Nuaillé, France) and 1% Penicillin–Streptomycin Solution (FUJIFILM Wako) at 37 °C with 5% CO_2_.

### 2.3. Plasmid Construction for Complete-Matched (CM) and Seed-Matched (SM) Luciferase Reporter Assays

The reporter plasmids were constructed using the psiCHECK-1 vector (Promega). Oligonucleotides used for insertion into the psiCHECK-1 vector were synthesized with *Xho*I or *EcoR*I, with the sticky end on both termini. Then, the synthesized oligonucleotides were inserted into the corresponding restriction enzyme sites, located at the 3′ UTR of the *Renilla luciferase* gene in the psiCHECK-1 vector. The plasmids containing CM sequences were synthesized to measure siRNA on-target activity. The plasmids containing three tandem repeats of SM sequences were synthesized to measure siRNA off-target activity. The sequences of the inserted oligonucleotides are shown in Supplementary Table S2.

### 2.4. Measurements of RNAi and Off-Target Activity by Dual Luciferase Reporter Assays

To perform the luciferase reporter assay, HeLa cells were inoculated in 24-well culture plates (1 × 10^5^ cells/well) for 24 h. The cells were simultaneously transfected with siRNA (0.05, 0.5, 5, or 50 nM), 100 ng of pGL3-Control vector (Promega), and 10 ng of the corresponding psiCHECK-1 vector, using Lipofectamine 2000 reagent (Thermo Fisher Scientific). The pGL3-Control vector encodes the firefly *luciferase* gene that was used as an internal control of luciferase activity. Control siRNA, siControl, does not target either CM- or SM-reporter constructs. At 24 h after transfection, cells were lysed by 1 × passive lysis buffer (Promega). Luciferase activity was measured by using the Dual-Luciferase Reporter Assay System (Promega) and GloMax Discover Microplate Reader (Promega). The on-target RNAi and off-target activity, via the transfection of each siRNA, was calculated from *Renilla* luciferase activity normalized by firefly luciferase activity, and presented as the relative percentage compared to the result of siControl.

### 2.5. Calculation of T_m_ Value Using Nearest-Neighbor Model

*T_m_* values were calculated for all possible regions in the siRNA duplex of 26 previously reported siRNAs [18] by means of the nearest-neighbor model [50]. *T_m_* values were calculated as follows.

$$T_{m}=\frac{1000\;\times \;\Delta H\;}{A+\Delta S+RIn\left(\frac{Ct}{4}\right)}-273.15+16.6log\left[{\mathrm{Na}}^{+}\right]$$

where Δ*H* is the sum of nearest neighbor enthalpy changes (kcal mol^−1^), *A* is the helix initiation constant (−10.8 cal mol^−1^K^−1^), Δ*S* is the sum of nearest neighbor entropy changes (kcal mol^−1^K^−1^), *R* is the gas constant (1.987 cal deg^−1^mol^−1^), *Ct* is total molecular concentration (100 µM), [Na^+^] is sodium ion concentration (100 mM). Enthalpy and entropy values in [50] were used for the calculation of *T_m_* values.

### 2.6. Determining the Responsible Regions by Random Sampling

Correlation heatmaps were generated in R Studio (ver. 3.4.0) to visualize the correlations between previously reported relative luciferase activities of the off-target effects of 26 siRNAs [18] and their *T_m_* values across all possible siRNA duplex regions. The lists of correlation coefficients are shown in Supplementary Table S3–S6. A sampling process was repeated to determine the statistically significant start and end positions of each responsible region. In each sampling cycle, 13 of 26 siRNAs were randomly sampled (10,400,600 possible combinations in total). The sampling process was repeated 1000 times. Among the selected samples, start (x) and end (y) positions of the top 1-ranked or top 10-ranked regions, with positive or negative correlations with luciferase activity, were recorded. Furthermore, the siRNA samples (*n* = 26) were randomly divided into training data (*n* = 13) and validation data (*n* = 13) (10,400,600 possible combinations in total), for a total of 1000 replications. In the training and validation data, correlations for each identified responsible region were randomly calculated and compared using a Student’s *t*-test. 

## 3. Results

### 3.1. Impacts of siRNA Base-Pairing Stabilities on on-Target and Off-Target Transcripts

**Figure 2:**
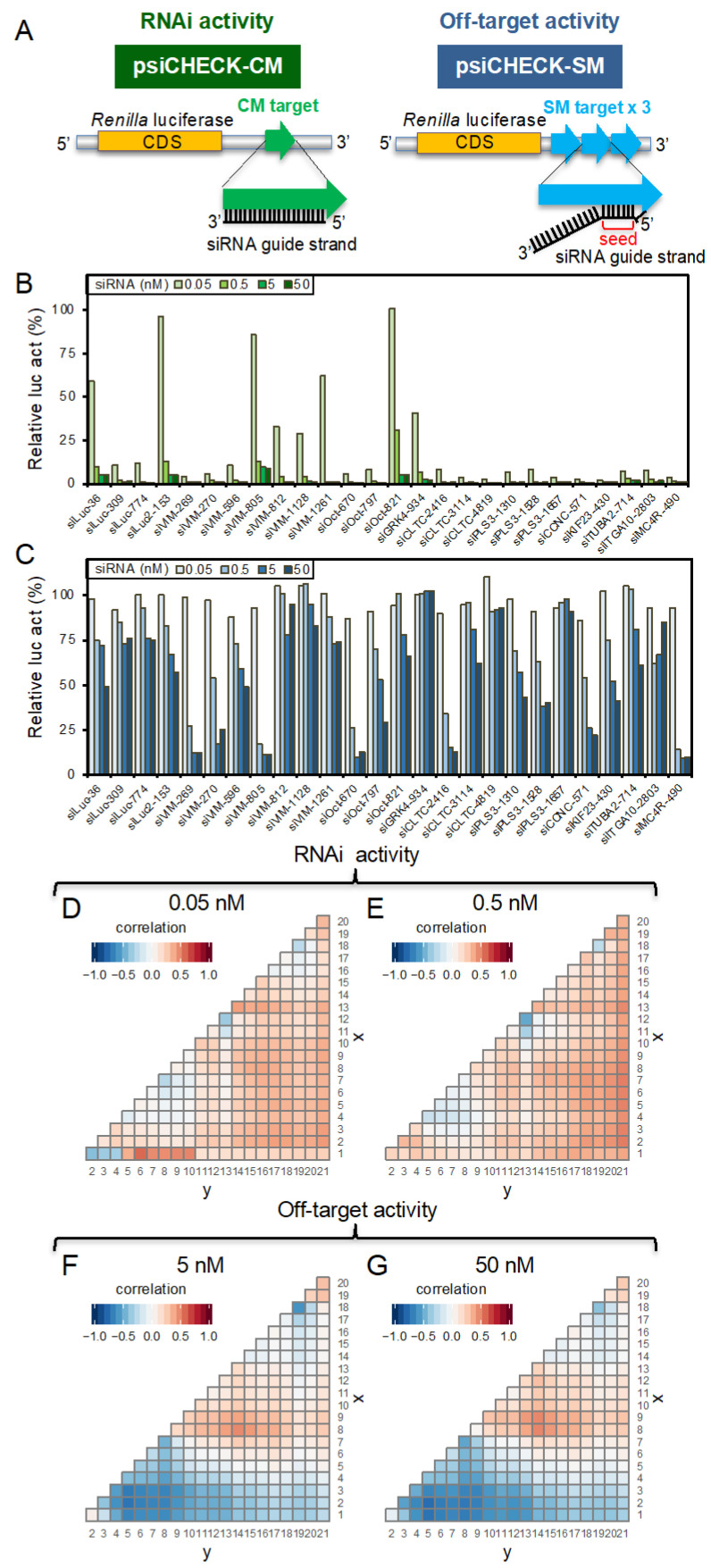
Previously reported on-target and off-target effects of 26 wild-type siRNAs measured using luciferase reporter assays [18], and the correlations of their activities with *T_m_* values at every siRNA subregion. (**A**) Schematic diagrams of reporter constructs used for on-target RNAi (left) and off-target (right) assays. The psiCHECK plasmids carrying the CM target of the siRNA guide strand in the 3′ UTR of the *Renilla luciferase* gene were used for on-target activity assays, and those carrying three tandem repeats of SM sequences were used for off-target activity assays. The on-target activities (**B**) and off-target activities (**C**) of 26 siRNAs were measured at 0.05, 0.5, 5, and 50 nM siRNA. The correlations between *T_m_* values of subsections of sequences within siRNA duplexes and the corresponding relative luciferase activities for CM on-target and SM off-target activities (**D**–**G**). The start position of each subsection is indicated on the vertical axis (x), and the end position is indicated on the horizontal axis (y). The position is numbered from 5′ end of the guide strand. Red indicates the positive correlation between *T_m_* value of each subsection and relative luciferase activity, indicating the negative correlation between *T_m_* value and on-target/off-target activity. Blue indicates the negative correlation between *T_m_* value of each subsection and relative luciferase activity, indicating the positive correlation between *T_m_* value and on-target/off-target activity.

To investigate the impact of base-pairing stabilities of all the possible subregions within siRNA on on-target RNAi and off-target activities, the data for on-target and off-target activities of 26 different siRNAs, shown in our previous reports, were used [18]. The data were obtained using reporter plasmids for each siRNA, which contain complete-matched (CM) or three tandem repeats of seed-matched (SM) target sequences in the 3′ UTRs of the *Renilla* luciferase gene in the psiCHECK vector (Figure 2A) [18,19]. The CM reporter was used for measuring on-target RNAi activity, and the SM reporter was used for measuring the seed-dependent off-target effect. The pGL3-Control vector expressing firefly luciferase was used as an internal control. The relative luciferase activity (*Renilla* luciferase activity/firefly luciferase activity) was measured in HeLa cells transfected with each siRNA and the corresponding *Renilla* luciferase reporter plasmid, with the firefly luciferase reporter plasmid used as an internal control. Low relative luciferase activity indicates high on-target or off-target activity. The siRNAs used for these assays satisfy the following functional siRNA sequence rules, as shown in Figure 1B: A or U residues at position 1 (5′ end of the guide strand), more than three A/U residues at nucleotide positions 2–7, G/C at position 19, and no long GC stretch [17].

All of the 26 siRNAs showed high on-target activities for CM targets, with less than 10% at 50 and 5 nM, and 30% at 0.5 nM (Figure 2B). However, the off-target activities measured using SM targets were much less effective, and the activities ranged from approximately 10 to 100% at 0.5, 5, and 50 nM siRNA concentrations, and almost no activities were observed at 0.05 nM (Figure 2C).

### 3.2. Identification of Responsible Subregions for RNAi and Off-Target Effects

Previously, we reported that the siRNA off-target effect is correlated with the thermodynamic stability of base-pairing between the siRNA seed region (nucleotides 2–8) and its off-target mRNAs [18]. However, the impacts of the thermodynamic stabilities of all possible nucleotide subregions within siRNA on off-target mRNAs are unknown. Then, in this study, we analyzed the effects of thermodynamic stabilities of every possible subregion within siRNA on CM on-target and SM off-target transcripts. The correlations between the relative luciferase activities for CM targets or SM targets and *T_m_* values at the corresponding siRNA subregions were calculated (Figure 2D–G). A low level of relative luciferase activity indicates a high level of on-target or off-target activity, and the *T_m_* values calculated using the nearest-neighbor method were used as indicators of thermodynamic stability: the RNA duplex with high *T_m_* values exhibits high base-pairing stability, that with low *T_m_* values has low stability.

The relative luciferase activities of 26 siRNAs on CM targets showed almost no strong correlation coefficients (−0.47 ≤ r ≤ 0.49) with *T_m_* values at any subregion, even at 0.05 nM siRNA, except for the positions 1–6, which showed slightly positive correlations (r = 0.57) (Figure 2D). These results suggest that the nucleotides at positions 1–6 are preferable to be A or U for high on-target activity, consistent with our sequence rules for the highly effective siRNA (Figure 1B) [17].

The results of the SM target assays at 0.05 and 0.5 nM siRNA concentrations showed weak off-target effects. Therefore, the results at both 5 nM and 50 nM were used for examining the correlations between the relative luciferase activities and *T_m_* values at the corresponding siRNA subregions. The results clearly reveal that at least two different regions are correlated with siRNA off-target activity. The *T_m_* values of nucleotides at 2–8 of the guide strand exhibited strong negative correlations with relative luciferase activities, indicating a positive correlation with off-target activity. The correlation coefficients at positions 2–5, 2–6, 2–7, and 2–8 were −0.76, −0.70, −0.67, and −0.71 at 50 nM siRNA, respectively (Figure 2G). In contrast, the positions 8–14 and 9–14 exhibited weak positive correlations (r = 0.49), indicating negative correlations with off-target activities. 

### 3.3. Identification of Positive and Negative Responsible Subregions for Off-Target Effect 

**Figure 3:**
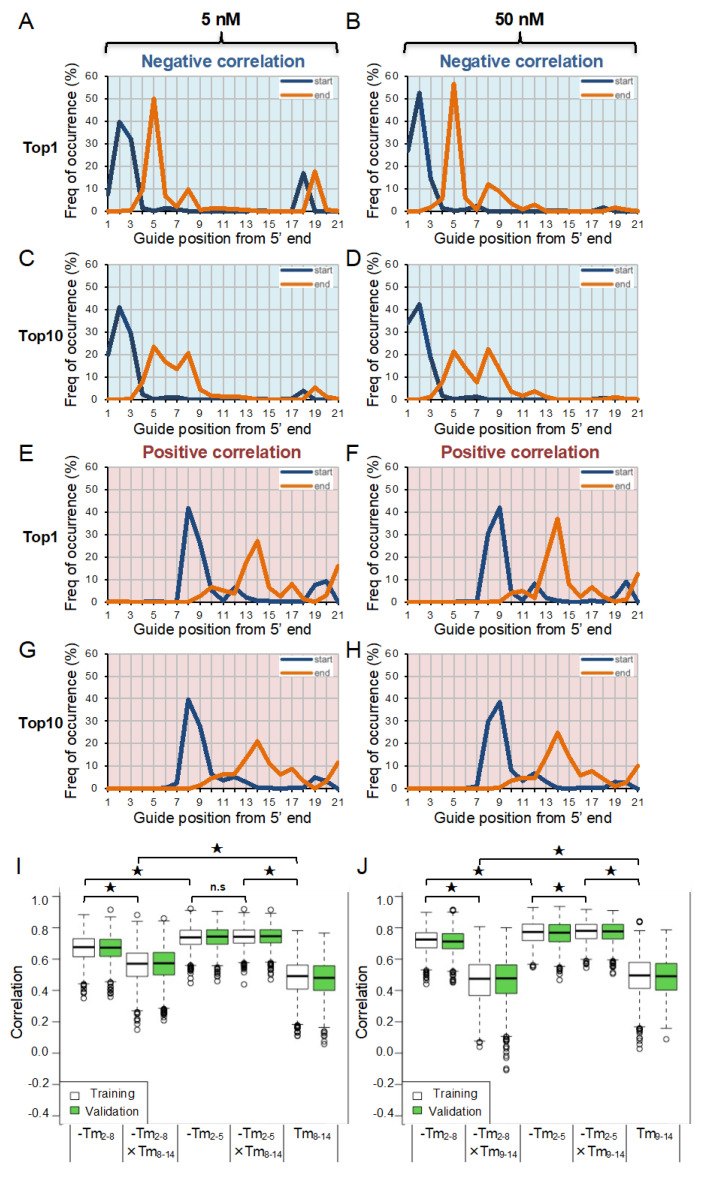
Machine learning technique to identify responsive regions for siRNA off-target effects. (**A**) Start (blue) and end (orange) positions of each responsive region significantly correlated with relative luciferase activities. Top 1-ranked positions with the most negative correlations at 5 nM (**A**) and 50 nM (**B**) siRNA, and top 10-ranked positions with the most negative correlations at 5 nM (**C**) and 50 nM (**D**) siRNA. Top 1-ranked positions with the most positive correlations at 5 nM (**E**) and 50 nM (**F**) siRNA, and top 10-ranked positions with the most positive correlations at 5 nM (**G**) and 50 nM (**H**). Sampling was repeated 1000 times for the calculation of each region. Mean values of correlation coefficients were calculated at 5 nM (**I**) and 50 nM (**J**) siRNA. In (**I**,**J**), white boxes indicate training data using randomly selected 13 samples, and green boxes indicate validation data using randomly selected 13 samples. The lines in the boxes indicate the median values. The lower and upper ends of each box are the 25% and 75% quartiles, respectively. The length of the bar is 1.5 times the size of the box. Points beyond these lines are outliers. Each *p*-value was determined by Student’s *t*-test (* *p* < 0.01). n.s., not significant. The training data and validation data were not significant for all combinations, not shown in the Figure.

To determine the exact start and end positions of each responsible subregion, a repeated random sampling process was performed on every possible combination of 13 siRNAs extracted from 26 siRNAs (Figure 3). In each sampling cycle, start (x) and end (y) positions of the subregions with the top 1-ranked, or the sum of the top 10-ranked, positively or negatively correlated positions were recorded for every 13 randomly sampled siRNAs. The random sampling process was repeated 1000 times to generate an x–y list determining the subregions with optimal correlations. The most frequent start and end positions (x–y) with the top 1-ranked negative correlation with relative luciferase activities were guide positions 2–5, at siRNA concentrations of both 5 nM (Figure 3A) and 50 nM (Figure 3B), whereas those with top 10-ranked correlations were subregions 2–5 and 2–8 (Figure 3C,D). By contrast, the most frequently occurring regions with top 1- and top 10-ranked positive correlations with relative luciferase activities were guide positions 8–14 at 5 nM siRNA (Figure 3E,G) and 9–14 at 50 nM siRNA (Figure 3F,H), respectively. The results were almost consistent with our previous study using microarray data, that the GC contents in target sequences corresponding to the nucleotides 8–15 are negatively correlated with off-target effects [51]. 

Subsequently, the absolute correlation coefficient for subregions 2–5, 2–8, and 8–14/9–14, at 5 nM (Figure 3I) and 50 nM (Figure 3J) siRNA concentration, was calculated. The relationships between relative luciferase activities and *T_m_* values at positions 2–5 were highly correlated (r = 0.77 at 50 nM, r = 0.73 at 5 nM) compared to those at positions 2–8 (r = 0.72 at 50 nM, r = 0.67 at 5 nM), based on the training data, and the correlation coefficients from the validation data were also higher at positions 2–5 (r = 0.76 at 50 nM, r = 0.74 at 5 nM) compared to those at positions 2–8 (r = 0.71 at 50 nM, r = 0.67 at 5 nM). These results suggest that the siRNA off-target effect is positively correlated with the thermodynamic stabilities at positions 2–5 and 2–8, although positions 2–5 have a greater impact on off-target effect compared to positions 2–8. The absolute correlation coefficient of positions 8–14 and 9–14 was also calculated. The correlation coefficient of the training data at positions 8–14 was 0.48, and that of the validation data was 0.47, at 5 nM siRNA (Figure 3I), and, respectively, 0.49 and 0.49 at 50 nM siRNA (Figure 3J). Thus, the absolute contribution of the thermodynamic properties of nucleotides 8–14/9–14 was revealed to be smaller compared to those at positions 2–5 or 2–8. However, the effect of the stabilities of nucleotides 2–5/2–8 and 8–14/9–14 on off-target effects were the opposite: the stable base-pairing of nucleotides 2–5 or 2-8 induces strong off-target effect, but the unstable base-pairing of nucleotides 8–14/9–14 induces strong off-target effect. Therefore, the integrated effects of 2–5/2–8 with 8–14/9–14 were calculated. The multiplied values of the correlation coefficients of subregions 2–5 with those of subregions 9–14 were slightly, but significantly, increased compared to subregions 2–5 alone, at 50 nM siRNA (Figure 3J), but unexpectedly the multiplied values of correlation coefficients of subregions 2–8 and 9–14 significantly decreased the correlations compared to subregion 2–8 alone. Similar results were also observed at 5 nM siRNA (Figure 3I). These results suggest that the appropriate combination of the thermodynamic stabilities of two different subregions 2–5 and 8–14/9–14 exhibits the highest impact on the siRNA off-target effect, although the effects of these two subregions are opposite. 

## 4. Discussion

In this study, we revealed that the off-target effect is regulated by the thermodynamic stabilities of two different siRNA regions with opposite effects: The lower stabilities of nucleotides 2–5 and the higher stabilities of nucleotides 8–14/9–14 were collaboratively acting to reduce the siRNA off-target effect (Figure 2). On the other hand, the thermodynamic stabilities of any subregions in the entire siRNA had minor impact on the on-target RNAi effect. This may be due to the fact that the slicer activity of the AGO2 protein is the primary determinant of RNAi activity, and that base-pairing stabilities are considered to exhibit little effect on RNAi activity.

In our previous report [18], we reported that *T_m_* values at positions 2–8 are strongly correlated with off-target effects. The same correlation was observed in the present study. However, the comprehensive analyses of the subregions within the entire siRNA revealed that the *T_m_* values at positions 2–5 showed significantly higher positive correlations with the off-target effect compared to those at positions 2–8 (Figure 3A–D), suggesting that the thermodynamic stabilities of nucleotides 6–8 have negligible impact on the off-target effects. These results are consistent with our previous study, that the siRNA seed region consists of two functionally different domains in response to 2′-OMe modifications [19]. The nucleotides 2–5 are stable and immobile on the quasi-helical surface of the AGO protein, both in single-stranded form and when base-paired with a target transcript [45,48,49,50,51]. Additionally, 2′-OMe modifications in the nucleotides 2–5 exhibited steric hindrance to reduce the off-target effect. Alternatively, the conformation of nucleotides 6–8 is flexible due to the interaction with the helix-7 domain of the AGO protein [45,46,47,48,49]. Furthermore, 2′-OMe modifications in the nucleotides 6–8 did not exhibit strong effects on either on-target or off-target activities, and rather enhanced both activities. The strong stability and immobility of nucleotides 2–5 on the AGO surface may be the main reason for the strong off-target effects, whereas the instability and flexibility of nucleotides 6–8 may be the cause of its weak contribution to the off-target effect.

**Figure 4:**
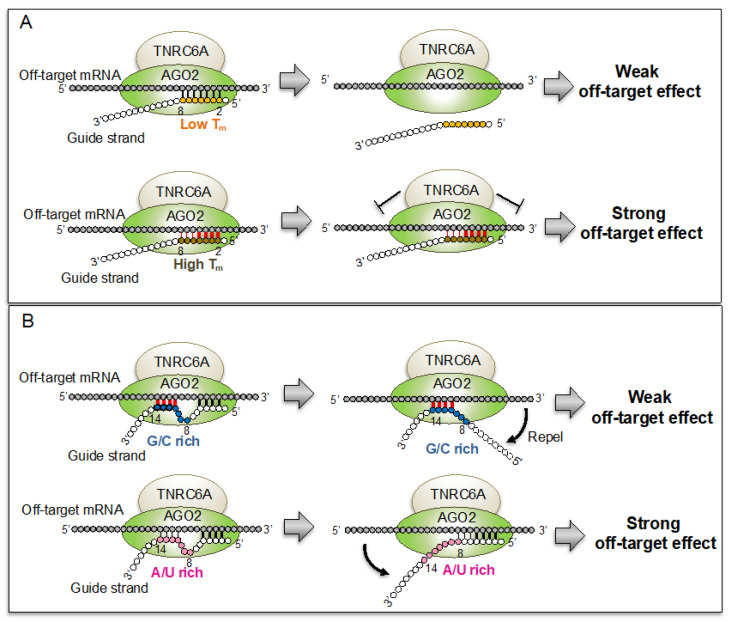
Regulation of off-target effects by thermodynamic stabilities or GC contents. (**A**) The effect of thermodynamic stability in the siRNA seed region. The siRNA with low *T_m_* value in the seed region showed weak off-target effect due to the weak base-pairing with off-target transcript (upper figure). The siRNA with high *T_m_* value in the seed region showed strong off-target effect due to the strong base-pairing with off-target transcript (lower figure). In the seed region, nucleotides 2–5 have stronger effects on the off-target transcript compared to nucleotides 2–8. (**B**) The effect of GC content in the siRNA non-seed positions, 8–14/9–14. The siRNA with high GC content in the non-seed region frequently form GC base-pairs with off-target transcript. This strong base-pairing is considered to repel the siRNA from the off-target transcript (upper figure). The siRNA with high AU content in the non-seed region frequently forms AU base-pairs with the off-target transcript. This weak base-pairing cannot repel the siRNA from off-target transcript (lower figure).

In this report, we clarified that the positions highly responsible for the off-target effect, in the seed region, are positions 2–5 (Figure 4A). In addition, the off-target effect is induced by siRNA with low *T_m_* values at positions 8–14/9–14 in the non-seed region (Figure 4B). These two different regions, with opposite effects, function synergistically on the off-target effect (Figure 3I,J). However, the integrated effects of 2–5 and 8–14/9–14 compared to those of 2–8 and 8–14/9–14 are apparently different: the multiplied values of the correlation coefficients of nucleotides 2–8 and 8–14/9–14 significantly lowered the correlations compared to those of nucleotides 2–8 (Figure 3J). In contrast, the multiplied values of the correlation coefficients of nucleotides 2–5 and 8–14/9–14 significantly increased the correlations compared to those of nucleotides 2–5. Although the non-seed region typically base-pairs with on-target transcripts, this region does not base-pair with off-target transcripts. Therefore, the high *T_m_* values of the non-seed region may represent the high GC content. When the GC content in the non-seed region is high, the nucleotides in the non-seed region are able to form GC-pairs with off-target transcripts randomly and at high frequencies (Figure 4B). The strong base-pairing of the GC-rich non-seed region with the off-target transcript is hypothesized to repel the siRNA from the transcript. However, the AU-rich weak base-pairing of the non-seed region might not remove the siRNA from the off-target transcript. In such processes, it is considered that the nucleotides 2–5, but not 2–8, can cooperatively act with nucleotides 8–14/9–14 (Figure 4B).

## 5. Conclusions

We clarified that the siRNA off-target effect is determined by base-pairing stabilities of two different regions with opposite effects: the off-target effect is induced by siRNA with high *T_m_* value at positions 2-5 in the seed region and the low *T_m_* value at positions 8–14/9–14 in the non-seed region. Furthermore, the integrated effects of the thermodynamic profiles of nucleotides 2–5 and 8–14/9–14 compared to those of nucleotides 2–8 and 8–14/9–14 are apparently different: the multiplied values of the correlation coefficients of nucleotides 2–5 and 8–14/9–14 significantly increased the absolute correlations with off-target effect compared to those of nucleotides 2–5 alone, but those of nucleotides 2–8 and 8–14/9–14 significantly decreased the absolute correlations compared to those of nucleotides 2-8. The strong base-pairing of the GC-rich non-seed region with the off-target transcript is hypothesized to repel the siRNA from the transcript to reduce off-target effect. In such repelling process, it is considered that the nucleotides 2–5, but not 2–8, can cooperatively act with nucleotides 8–14/9–14.

## Supplementary Materials

The following are available online at https://www.mdpi.com/article/10.3390/genes13020319/s1, Table S1. siRNA sequences; Table S2. Target sequences in psiCHECK-CM and psiCHECK-SM reporters; Table S3. Correlation coefficients between RNAi activities at 0.05 nM and *Tm* values in the siRNA subregions; Table S4. Correlation coefficients between RNAi activities at 0.5 nM and *T_m_* values in the siRNA subregions; Table S5. Correlation coefficients between off-target activities at 5 nM and *T_m_* values in the siRNA subregions; Table S6. Correlation coefficients between off-target activities at 50 nM and *T_m_* values in the siRNA subregions.
