# Supplementary material for: The siRNA Off-Target Effect Is Determined by Base-Pairing Stabilities of Two Different Regions with Opposite Effects
Source: Genes (Basel). 2022 Feb 9;13(2):319. doi: 10.3390/genes13020319 (PMC8872465; doi:10.3390/genes13020319)
Supplement: Supplementary file 1 [file genes-13-00319-s001.zip › genes-1598245-supplementary/Genes_Supple-table_S1-S2_Kobayashi et al.pdf]

Supplementary Table S1. siRNA sequences.

siRNA	Passenger strand (5'→3')	Guide strand (5'→3')
siControl	GCCACAACGUCUAUAUCAUGG	AUGAUAUAGACGUUGUGGCUG
siLuc-36	CAUUCUAUCCGCUGGAAGAUG	UCUUCCAGCGGAUAGAAUGGC
siLuc-309	CCGCGAACGACAUUUAUAAUG	UUAUAAAUGUCGUUCGCGGGC
siLuc-774	GAUUUCGAGUCGUCUUAAUGU	AUUAAGACGACUCGAAAUCCA
siLuc2-153	CGUACGCGGAAUACUUCGAUU	UCGAAGUAUUCCGCGUACGUG
siVIM-269	GCCAUCAACACCGAGUUCAAG	UGAACUCGGUGUUGAUGGCGU
siVIM-270	CCAUCAACACCGAGUUCAAGA	UUGAACUCGGUGUUGAUGGCG
siVIM-596	GAAAACACCCUGCAAUCUUUC	AAGAUUGCAGGGUGUUUUCGG
siVIM-805	GCGUGACGUACGUCAGCAAUA	UUGCUGACGUACGUCACGCAG
siVIM-812	GUACGUCAGCAAUAUGAAAGU	UUUCAUAUUGCUGACGUACGU
siVIM-1128	CUCGUCACCUUCGUGAAUACC	UAUUCACGAAGGUGACGAGCC
siVIM-1261	GAACCUGAGGGAAACUAAUCU	AUUAGUUUCCCUCAGGUUCAG
siOCT-670	GAGAAAGCGAACUAGCAUUGA	AAUGCUAGUUCGCUUUCUCUU
siOCT-797	GUUCGAGUAUGGUUCUGUAAC	UACAGAACCAUACUCGAACCA
siOCT-821	CGCCAGAAGGGCAAAAGAUCA	AUCUUUUGCCCUUCUGGCGCC
siGRK4-934	CUUGAAGCCUGAGAAUAUUCU	AAUAUUCUCAGGCUUCAAGUC
siCLTC-2416	GGUGAAUCCAAGUCGACUUCC	AAGUCGACUUGGAUUCACCUU
siCLTC-3114	GUGUUAUGGAGUAUAUUAACC	UUAAUAUACUCCAUAACACGU
siCLTC-4819	GACAAAGGUGGAUAAAUUAGA	UAAUUUAUCCACCUUUGUCAA
siPLS3-1310	GUUCCUGUUGACUGGAGUAAG	UACUCCAGUCAACAGGAACUU
siPLS3-1528	GGAAGAUCUUGGAGAUGGUCA	ACCAUCUCCAAGAUCUUCCAG
siPLS3-1657	GGCAGUUGUGGAUUUAAUUGA	AAUUAAAUCCACAACUGCCAA
siCCNC-571	GUAUCCUCCUUUCAUGAUAGC	UAUCAUGAAAGGAGGAUACAG
siKIF23-430	GUCAUUUCAAGCUAAACGAUA	UCGUUUAGCUUGAAAUGACCC
siTUBA2-714	CGGCCUCCCUGCGAUUUGACG	UCAAAUCGCAGGGAGGCCGUG
siITGA10-2803	CCUCAGCCUACAUCCAAUAUG	UAUUGGAUGUAGGCUGAGGUC
siMC4R-490	GCGGGUUGGGAUCAUCAUAAG	UAUGAUGAUCCCAACCCGCUU

Note: The number following each siRNA name indicates the nucleotide position in each target gene corresponding to the 3´ end of each siRNA guide strand.

Luc = Firefly Luciferase, VIM = Vimentin, OCT = Oct4 transcription factor, GRK4 = G protein-coupled receptor kinase 4, CLTC = Clathrin heavy chain, PLS3 = Plastin 3, CCNC = Cyclin C, KIF23 = Kinesin family member 23, TUBA2 = tubulin alpha 2, ITGA10 = integrin alpha 10, MC4R = Melanocortin 4 receptor.

Supplementary Table S2. Target sequences in psiCHECK-CM and psiCHECK-SM reporters.

Target sequences in psiCHECK-CM.

Oligonucleotide name	Sequence (5'→3')
Luc-36-cm	GCCAUUCUAUCCGCUGGAAGAUG
Luc-309-cm	GCCCGCGAACGACAUUUAUAAUG
Luc-774-cm	UGGAUUUCGAGUCGUCUUAAUGU
Luc2-153-cm	CACGUACGCGGAAUACUUCGAAA
VIM-269-cm	ACGCCAUCAACACCGAGUUCAAG
VIM-270-cm	CGCCAUCAACACCGAGUUCAAGA
VIM-596-cm	CCGAAAACACCCUGCAAUCUUUC
VIM-805-cm	CUGCGUGACGUACGUCAGCAAUA
VIM-812-cm	ACGUACGUCAGCAAUAUGAAAGU
VIM-1128-cm	GGCUCGUCACCUUCGUGAAUACC
VIM-1261-cm	CUGAACCUGAGGGAAACUAAUCU
OCT-670-cm	AAGAGAAAGCGAACUAGCAUUGA
OCT-797-cm	UGGUUCGAGUAUGGUUCUGUAAC
OCT-821-cm	GGCGCCAGAAGGGCAAAAGAUCA
GRK4-934-cm	GACUUGAAGCCUGAGAAUAUUCU
CLTC-2416-cm	AAGGUGAAUCCAAGUCGACUUCC
CLTC-3114-cm	ACGUGUUAUGGAGUAUAUUAACC
CLTC-4819-cm	UUGACAAAGGUGGAUAAAUUAGA
PLS3-1310-cm	AAGUUCCUGUUGACUGGAGUAAG
PLS3-1528-cm	CUGGAAGAUCUUGGAGAUGGUCA
PLS3-1657-cm	UUGGCAGUUGUGGAUUUAAUUGA
CCNC-571-cm	CUGUAUCCUCCUUUCAUGAUAGC
KIF23-430-cm	GGGUCAUUUCAAGCUAAACGAUA
TUBA2-714-cm	CACGGCCUCCCUGCGAUUUGACG
ITGA10-2803-cm	GACCUCAGCCUACAUCCAAUAUG
MC4R-490-cm	AAGCGGGUUGGGAUCAUCAUAAG

Target sequences in psiCHECK-SM.

Oligonucleotide name	Sequence (5'→3')
Luc-36-sm	CUUUGGAUGAAACCUGGAAGAUG

Luc-309-sm	ACUAACCCCUAAUAUUUAUAAUG
Luc-774-sm	UAGGAUGCCAUCGGUCUUAAUGU
Luc2-153-sm	AAGCGGUGCCAAGUACUUCGAAA
VIM-269-sm	CAAUGAUGCACCAGGAGUUCAAG
VIM-270-sm	AAGGAAGGGAGAGGAGUUCAAGA
VIM-596-sm	CGUUACUCUCGGAGCAAUCUUUC
VIM-805-sm	GGUGCCCAAGGAAGUCAGCAAUA
VIM-812-sm	CUAGGUGAAACUUAUAUGAAAGU
VIM-1128-sm	GAGGCAAAUGACGCGUGAAUACC
VIM-1261-sm	CAUGUCAUUACACAAACUAAUCU
OCT-670-sm	UACCUACACUAAUCUAGCAUUGA
OCT-797-sm	GCUGAAGCUAGUAGUUCUGUAAC
OCT-821-sm	CACUGAGCGGUUUCAAAAGAUCA
GRK4-934-sm	AGGCAAGCGGCGAAGAAUAUUCU
CLTC-2416-sm	CCGAGCAAGUGGUGUCGACUUCC
CLTC-3114-sm	CACUUGCCACUCAUAUAUUAACC
CLTC-4819-sm	ACUUGCUCGAGUCAUAAAUUAGA
PLS3-1310-sm	GCCAAUAUCGAUCCUGGAGUAAG
PLS3-1528-sm	GGUUAUCUCUUCCGAGAUGGUCA
PLS3-1657-sm	UCGUGCUGAAAGUAUUUAAUUGA
CCNC-571-sm	GGUCUUCUCUCGAUCAUGAUAGC
KIF23-430-sm	GAAUGCGCAACACCUAAACGAUA
TUBA2-714-sm	CUAUGACCGGCACCGAUUUGACG
ITGA10-2803-sm	ACGUAUGAACUGGAUCCAAUAUG
MC4R-490-sm	CAUCCUCUGGUUUUCAUCAUAAG
